# Biotransformation of the Fluoroquinolone Antibiotic, Levofloxacin, by the Free and Immobilized Secretome of *Coriolopsis gallica*

**DOI:** 10.3390/jof10120861

**Published:** 2024-12-12

**Authors:** Karima Staita, Marwa Khmaissa, Imen Akrout, Stéphane Greff, Bouthaina Ghariani, Annick Turbé-Doan, Julien Lambert, Anne Lomascolo, Quentin Albert, Craig B. Faulds, Giuliano Sciara, Héla Zouari-Mechichi, Eric Record, Tahar Mechichi

**Affiliations:** 1 Laboratoire de Biochimie et de Génie Enzymatique des Lipases, Ecole Nationale d’Ingénieurs de Sfax, Université de Sfax, Sfax 3038, Tunisia; karima.staita@enis.tn (K.S.); marwadjmel@gmail.com (M.K.); imen.akrout@enis.tn (I.A.); bouthaina.ghar@live.fr (B.G.); hela.zouari@isbs.usf.tn (H.Z.-M.); 2 INRAE, Aix Marseille Univ BBF, Biodiversité et Biotechnologie Fongiques, 13288 Marseille, France; annick.doan@univ-amu.fr (A.T.-D.); julien.lambert@inrae.fr (J.L.); anne.lomascolo@univ-amu.fr (A.L.); quentin.albert@univ-amu.fr (Q.A.); craig.faulds@univ-amu.fr (C.B.F.); giuliano.sciara@inrae.fr (G.S.); 3 IMBE, UMR 7263, CNRS, IRD, Aix-Marseille Université, Avignon Université, Station Marine d’Endoume, Rue de la Batterie des Lions, 13007 Marseille, France; stephane.greff@imbe.fr

**Keywords:** fluoroquinolone, levofloxacin, *Coriolopsis gallica* secretome, biotransformation, mediator, alginate immobilization, fungal bioremediation

## Abstract

Antibiotics play a crucial role in human and animal medical healthcare, but widespread use and overuse of antibiotics poses alarming health and environmental issues. Fluoroquinolones constitute a class of antibiotics that has already become ubiquitous in the environment, and their increasing use and high persistence prompt growing concern. Here we investigated a fungal secretome prepared from the white-rot fungus *Coriolopsis gallica*, which is able to effectively degrade the environmentally persistent fluoroquinolone, levofloxacin. We tested various physical–chemical factors such as concentrations of 1-hydroxybenzotriazol (HBT), of enzyme, and of antibiotic, and pH and temperature of the reaction for biotransformation of the antibiotic. We compared the free with the immobilized *Coriolopsis gallica* secretome proteins, and analyzed the collective reaction products for residual activity against *E. coli* (growth inhibition test). We also performed HPLC analysis. The results show that treatment with the free secretome yielded a highest removal efficiency of 50 mg L^−1^ levofloxacin in the presence of 2.5 mM HBT, whereas the immobilized secretome was only able to degrade 10 mg L^−1^ levofloxacin with the same concentration of mediator, but presenting the advantage of being reusable.

## 1. Introduction

Fluoroquinolone antibiotics have become a subject of growing concern, due to their increasing use in households, hospitals, and veterinary applications [1,2,3], with dosage concentrations ranging from ng L^−1^ to µg L^−1^ [4,5,6]. Fluoroquinolones are characterized by broad-spectrum antimicrobial activity, strong bactericidal activity, and a high oral absorption efficiency [7]. They are recognized as critically important ‘drug of choice’ antimicrobial compounds [8,9], but they are also considered pseudo-persistent compounds, as up to 70% of them are excreted in a non-metabolized form, which will possibly promote further antibiotic resistance in the environment [10]. The low biotransformation rate of fluoroquinolones makes conventional water treatments ineffective or carry drawbacks [11]. Many studies have focused on eliminating these antibiotics via various physical–chemical methods such as membrane separation techniques [12], adsorption on activated carbon [13], advanced oxidation [13], ozone and electrochemical advanced oxidation filtration, and chemical flocculation [14]. Unfortunately, some of these treatments are not economically sustainable, while others are not feasible for large-scale application [15]. The sludge generated in treatment plants creates significant contamination, requiring further costly regeneration and post-treatment operations on the solid wastes [16,17]. As a sustainable alternative, biological treatment, or ‘bioremediation’, holds great promise as a cost-effective, innovative technology that employs microorganisms or microbial enzymes to degrade pollutants and transform them into less toxic/non-toxic products [18,19,20].

The ability of filamentous fungi to produce several enzymes and secondary metabolites makes them valuable tools for bioremediation processes, especially for the removal of recalcitrant pollutants [21,22]. Within the fungal kingdom, white-rot fungi are a potentially powerful group of fungi known to be able to decompose various compounds [23]. For instance, these organisms have the potential to adsorb [24], transform [25,26], and even decolorize [27,28] a large spectrum of industrial dyes via non-specific extracellular enzymatic systems, utilizing lignin-modifying oxidoreductases such as laccases (Lac, E.C. 1.10.3.2; AA1), lignin peroxidase (LiP, E.C. 1.11.1.14; AA2), manganese peroxidase (MnP, E.C. 1.11.1.13; AA2), and versatile peroxidase (VP, E.C. 1.11.1.16; AA2), and intracellular enzymes such as cytochrome P450 systems [24,29]. For example, *Irpex lacteus*, *Trametes versicolor*, *Porostereum spadiceum*, *Bjerkandera adusta*, and *Coriolopsis gallica* have demonstrated the ability to biotransform the fluoroquinolone antibiotics ciprofloxacin, norfloxacin, ofloxacin, and levofloxacin [24,30,31,32]. The laccase produced by *Trametes polyzona* was reported to degrade quinolone antibiotics via a redox mediator-free system [33]. Furthermore, regarding the redox potential of the treated antibiotics, the use of a redox mediator that is able to act as electron shuttle between the enzyme and the substrate is required to achieve efficient levels of biotransformation [34,35].

Various enzyme immobilization strategies have been previously employed, (i) to reduce the cost of enzymes production used for the bioremediation of pharmaceutical compounds, (ii) to make them more widely affordable and to increase their reuse, and (iii) to improve their stability [36,37]. There has been extensive research into laccase immobilization using various methods and supports [38,39,40,41]. Among the different approaches, entrapment is considered a good choice as it is an easy process to prepare and causes relatively little damage to the native enzyme structure [42]. Entrapment in calcium alginate gel offers a number of advantages due to its simplicity, superior chemical stability, ecology-consciousness, and cost-effectiveness [43,44]. Yang et al. (2017) immobilized laccases from *Cerrena unicolor* to biotransform six antibiotics with or without mediator, on 2,2′-azino-bis(3-ethylbenzothiazoline-6-sulfonic acid) as substrate (ABTS) [35]. Additionally, Becker et al. (2016) evaluated the biotransformation of 38 antibiotics of different classes in an enzymatic membrane bioreactor by immobilizing laccases produced by *T. versicolor* [45].

In previous work [32], we studied the biotransformation of the fluoroquinolone levofloxacin by three fungi, i.e., *C. gallica* CLBE55, *Thielavia* sp. HJ22, and *Thielavia* sp. CH1, and demonstrated that *C. gallica* was the most efficient strain for this reaction. Based on activity assays and proteomics analysis, we proposed that laccases and dye-decolorizing peroxidases (DyP) of *C. gallica* could be the primary biocatalysts and drivers involved in the enzymatic biotransformation of levofloxacin. However, this does not rule out the role of other secreted proteins in the biotransformation. This study aims to optimize the efficiency of levofloxacin biotransformation by the secretome of *Coriolopsis gallica*, evaluating the impact of physicochemical parameters and comparing free and immobilized systems by using the free and immobilized culture cell-free secretome of *C. gallica* in the presence of the chemical mediator 1-hydroxybenzotriazole (HBT). HBT was selected as a chemical mediator as it emerged in previous study as the most effective mediator for various applications [27,28,41]. HBT has been shown to effectively facilitate electron transfer, thereby improving enzymatic activity, whereas at high concentrations, inhibitory effects have been already observed, potentially due to competitive binding or alterations in the enzyme’s active site. By elucidating these underlying mechanisms, we aimed to provide a clearer understanding of its role in the antibiotic biotransformation process. We also studied the influence of several other physical–chemical factors on the biotransformation of levofloxacin, including the antibiotic or enzyme concentrations. The products of the biotransformation were also analyzed, which provided data confirming that the laccases of *C. gallica* secretome are the main actors of the biotransformation.

## 2. Materials and Methods

### 2.1. Chemicals

2,6-dimethoxyphenol (DMP), 1-hydroxybenzotriazole (HBT), copper sulfate (CuSO_4_), levofloxacin, and other products were obtained from Sigma-Aldrich (Saint-Quentin-Fallavier, France).

### 2.2. Fungal Strain and Culture Conditions

The strain used in this study, *C. gallica* BS9, was isolated from a Tunisian forest biotope near Bousalem in Northwestern Tunisia in 2008 (GPS coordinates: 36.653681, 8.904576) [32].

Solid cultures of strain BS9 were performed on potato dextrose agar (PDA) medium, with 39 g of dehydrated medium suspended in 1000 mL of distilled water and sterilized by autoclaving at 120 °C for 30 min.

Liquid precultures were performed in 100 mL of M7 medium. This basal medium contained (per L): glucose (100 g), soya peptone (5 g), yeast extract (1 g), ammonium citrate (2 g), MgSO_4_ (0.5 g), K_2_HPO_4_ (1 g), KCl (0.5 g), and trace element solution (1 mL). The composition of the trace element solution was (g L^−1^): B_4_O_7_Na_2_ 10H_2_O (0.1), CuSO_4_ 5H_2_O (0.01), FeSO_4_ 7H_2_O (0.05), MnSO_4_ 4H_2_O (0.01), ZnSO_7_ H_2_O (0.07), and MoNH_4_ 4H_2_O (0.01) [46]. Inoculation was carried out in 250 mL Erlenmeyer flasks by adding three 6 mm-diameter agar plugs from an actively growing fungus on potato dextrose agar (PDA). The flasks were incubated at 30 °C for three days at 160 rpm. The mycelium was then homogenized using glass beads and used to inoculate 500 mL Erlenmeyer flasks containing 100 mL of M7 medium at pH 5.5 supplemented with (300 µM) CuSO_4_as laccase inducer. Cultures were incubated at 30 °C at 160 rpm.

### 2.3. Production of Coriolopsis gallica Secretome

The production of *C. gallica* secretome was carried out in 500 mL Erlenmeyer flasks containing 100 mL of M7 liquid culture medium in the presence of CuSO_4_ (300 μM) to induce laccase production. Enzyme activity was measured every day for 14 days. The culture medium was then filtered (through 0.45 µm) in order to collect the cell-free secretome.

The filtered secretome was concentrated using a dialysis tubing (12 kDa, Sigma-Aldrich, Saint Quentin Fallavier, France) placed in polyethylene glycol (PEG 10,000) until the desired volume was reached. Then, laccase activity was measured, and the concentrated secretome was stored at −20 °C until further experiments.

### 2.4. Purification of Laccase

The 10-day-culture medium of *C. gallica* was separated from mycelia by filtration on Whatman paper. The filtered supernatant was then concentrated inside a dialysis tube (12 kDa) covered with PEG (10,000 Da) for 20 h at 4 °C. The concentrated supernatant was brought to 80% (*w*/*v*) saturation with solid ammonium sulfate at 4 °C. A protein precipitate was recovered by centrifugation at 12,000× *g* for 20 min at 4 °C and resuspended in sodium tartrate buffer (20 mM pH 5.5). The protein solution was applied to a PD-10 column (Sigma-Aldrich, Saint-Quentin-Fallavier, France) previously equilibrated with the same buffer. The enzyme solution was applied to a Q-Sepharose chromatography (2 × 30 cm) (Sigma-Aldrich) pre-equilibrated with sodium tartrate buffer (pH 5.5). To elute the proteins, we applied a linear concentration gradient of NaCl from 0 to 500 mM (in 100 mL of sodium tartrate buffer) at a flow rate of 30 mL h^−1^.

### 2.5. Determination of Laccase Activity

Laccase activity is determined based on the rate of oxidation of 2,6-dimethylphenol (DMP). The appearance of the product was measured at λ = 469 nm (ɛ = 27,500 M^−1^ cm^−1^). The reaction mixture contained 100 µL of 50 mM DMP, 500 μL of 50 mM citrate buffer (pH 5.5), 50 µL of enzyme solution, and 350 μL of distilled water. One unit of laccase activity (U) was defined as the amount of enzyme required to catalyze the oxidation of 1 µmol mL^−1^ of substrate per minute. Each assay was performed in triplicate.

### 2.6. Immobilization of Coriolopsis gallica Proteins

The immobilization method used in this work was based on the inclusion of secretome proteins in alginate beads [41]. The protein suspension was entrapped in alginate gel. A volume of 100 mL of alginate solution (2% *w*/*v*) was prepared by dissolving sodium alginate in distilled water. Then, the secretome proteins were added and mixed with the previously prepared alginate solution. The mixture was injected into a CaCl_2_ solution (2% *w*/*v*) under stirring. The beads formed were left for one hour in the calcium chloride solution under very moderate agitation. After one hour, the calcium alginate beads were collected from the solution and washed twice with distilled water. The washed solutions were collected to calculate the percentage of immobilization, per the following formula:

$$Immobilization\;\%=(\frac{\left(Ai-A_{wash}\right)}{Ai})\times 100$$

where Ai is initial activity of the free enzyme introduced into the Ca alginate solution mixture assayed using DMP as substrate, and A_wash_ is the laccase activity detected in the three wash solutions assayed using the same substrate. All the beads were kept in a sterile tube at 4 °C until further use.

### 2.7. Determination of the Percentage of Levofloxacin Biotransformation

The percentage of antibiotic biotransformation was determined by the agar diffusion method as follows. A fresh culture of *E. coli* was used as a control strain and was spread on Petri dishes containing a Muller–Hinton agar (MHA) medium, i.e., 17.5 g L^−1^ peptone, 2 g L^−1^ meat extract, 1.5 g L^−1^ starch, and 17 g L^−1^ agar, at pH 7.3. The culture was spread using sterile cotton swabs, and excess fluid was removed by turning the swab against the inside of the tube to avoid over-inoculation of plates.

The bacterial suspension used to inoculate the Petri dishes was prepared by selecting a variety of morphologically similar *E. coli* colonies on Luria Bertaini (LB) broth (yeast extract 5.0 g L^−1^, tryptone 10.0 g L^−1^, NaCl 10.0 g L^−1^) for 1 h at 37 °C under stirring at 200 rpm. These colonies were then suspended in a sterile saline solution (0.85% NaCl *w*/*v* in water) to the density of a McFarland 0.5 standard approximately corresponding to an absorbance reading of 0.08–0.1 at 625 nm.

Then, a sterile cotton swab was immersed in the inoculum suspension, and the excess fluid was eliminated by swiping the swab against the inside of the tube to avoid over-inoculation of plates. Then, 50 μL of the sample and the chemical control were placed in a 6 mm circular well of the Petri dish. Plates were then incubated at 37 °C for 24 h. The experiment was conducted in duplicate, and the diameters of *E. coli* inhibition zones obtained after incubation were measured by a ruler on the surface of the Petri dish.

Percentage of biotransformation was determined according to diameter of the inhibition zone, as follows:

$$Biotransformation\;\%=(\frac{\left(D_{t0}-D_{tf}\right)}{D_{t0}})\times 100$$

where D_t0_ is the diameter measured at time 0, and D_tf_ is the diameter at 24 h of incubation.

### 2.8. Determination of the Effect of Physical–Chemical Factors on Levofloxacin Biotransformation by the Coriolopsis gallica Secretome Proteins

The effect of physical–chemical factors on the biotransformation of levofloxacin by free and immobilized secretome proteins was studied through a series of reactions in which one of the following parameters was varied each time: levofloxacin concentration, HBT mediator concentration, protein concentration, and effect of pH and temperature. The initial laccase-like activity used was 5 U mL^−1^. We selected the various values based on previous studies in the literature, which provided a foundation for our choices. Additionally, we aimed to broaden the range of values to explore a wider spectrum of conditions and their effects on biotransformation. This approach allows us to gather more comprehensive data and insights.

The effect of levofloxacin concentration on biotransformation was tested in 5 mL glass tubes on a 2 mL reaction volume by incubating the secretome at different antibiotic concentrations (10, 20, 30, 40, 50, 60, 70, 80, 90, and 100 mg L^−1^) in the presence of 5 U mL^−1^ of *C. gallica* secretome, 5 mM HBT mediator, and sodium citrate buffer (100 mM, pH 5.0). A control-reaction mixture was run without secretome. The reaction mixtures were incubated at 30 °C for 24 h.

The effect of HBT concentration on the biotransformation of levofloxacin was tested under the same conditions at increasing HBT concentrations, i.e., 1.25, 2.5, 5, 7.5, and 10 mM. The effect of secretome concentration was studied at increasing concentrations, i.e., 1.25, 2.5, 5, 7.5, and 10 U mL^−1^. The effect of temperature was determined by incubating the reaction medium in a range of temperatures, i.e., 30 °C, 40 °C, and 50 °C. The effect of pH was determined by incubating the reaction medium in a range of pH values from 3.0 to 7.0 using the following buffers: sodium-citrate buffer (100 mM) for pH 2.0 and 3.0, tartrate buffer (100 mM) for pH 4.0 and 5.0, and citrate–phosphate buffer (100 mM) for pH 6.0 and 7.0.

### 2.9. UHPLC–UV and HR-MS Analyses of Levofloxacin in the Presence of Coriolopsis gallica Secretome or Laccase for the Dereplication of Levofloxacin Degradation Products

Liquid chromatography analyses (UHPLC) coupled with high-resolution mass spectrometry (HR-MS) were performed on *C. gallica* secretome extracts, or laccase, supplemented with levofloxacin according to the same analysis protocol used in Ben Ayed et al. (2022, 2024) [25,32]. The samples containing the secretome of *C. gallica*, or the reaction mixture with laccase, were extracted with 100 mL ethyl acetate supplemented with 0.001% acetic acid. Then, extracts were evaporated to dryness in a rotary evaporator at 50 °C with a speed of 100 rpm. The dry residues were dissolved in 2 mL methanol, then vortexed for 30 s, after which 800 µL of these extracts was transferred to 2 mL vials and analyzed by UHPLC. The comparative UHPLC–UV–MS analyses were performed in triplicates. UV chromatograms were acquired at 280 and 325 nm. Data-dependent MS/MS acquisitions were also performed on the 3 major features from 50 to 1200 amu at 8 Hz in positive mode with a collision energy of 25–50 eV (50% of the collision time at each energy). Each MS1 and MS2 spectrum was manually analyzed and annotated for the dereplication of the levofloxacin degradation products.

## 3. Results

### 3.1. Production of Coriolopsis gallica Secretome

**Figure 1:**
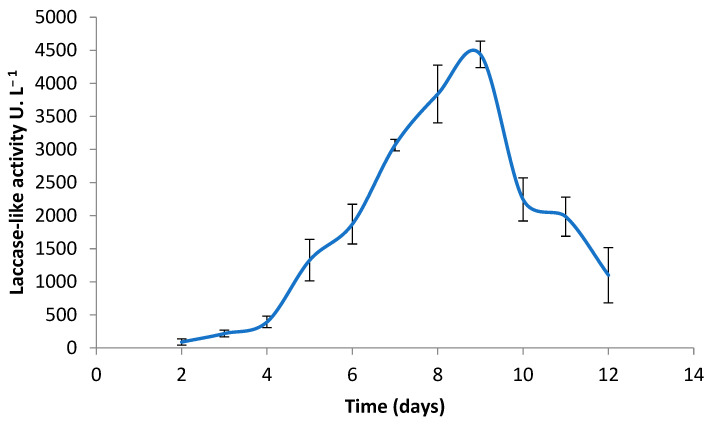
Laccase-like activity of *C. gallica* produced in M7 medium supplemented with 300 µM CuSO_4_. The error bars represent the standard deviation from the mean of the measured parameter across three experimental replicates.

*C. gallica* was grown on a synthetic medium, and the laccase-like activity of the secretome was measured for 14 days. A laccase-like activity was detected from day 5 of incubation (Figure 1) with an estimated average activity of 441 U L^−1^. This laccase-like enzyme production increased progressively, reaching an optimum at day 9 of 4500 U L^−1^, and then decreased gradually from day 10. This decrease was probably due to proteases that degrade laccases and other secreted proteins, causing their inactivation and denaturation. For further experiments, the cell-free supernatant was collected at day 9 and used as the biocatalysts for the levofloxacin biotransformation.

### 3.2. Purification of Laccase

A laccase was purified from the culture medium of *C. gallica* (*Cga*Lac1) supplemented with CuSO_4_ (300 µM) by a one-step chromatography column using Q-Sepharose anion chromatography. Table 1 summarizes the purification process of *Cga*Lac1 (as activity assayed against DMP). With respect to the purification process, *Cga*Lac1 was purified 5-fold with a yield of 18.3%.

### 3.3. Laccase Immobilization into Alginate Beads

The preparation of beads with the requisite stiffness and permeability for enzyme gel entrapment is based on both the concentration of sodium alginate (gelling agent) and the concentration of calcium ions (cross-linking agent) in the solution. The immobilization yield (ratio of the specific activity of entrapped enzymes to the specific activity of free enzymes) was determined using the equation described in Section 2.6. The optimal conditions were 2% (*w*/*v*) sodium alginate and 2% (*w*/*v*) CaCl_2_ (Daâssi et al., 2014) [41]. Under these conditions, immobilization yield of laccase activity was 98 ± 4.7%.

### 3.4. Effect of Physical–Chemical Factors on Levofloxacin Biotransformation by Free and Immobilized C. gallica Secretome

#### 3.4.1. Effect of Levofloxacin Concentration

**Figure 2:**
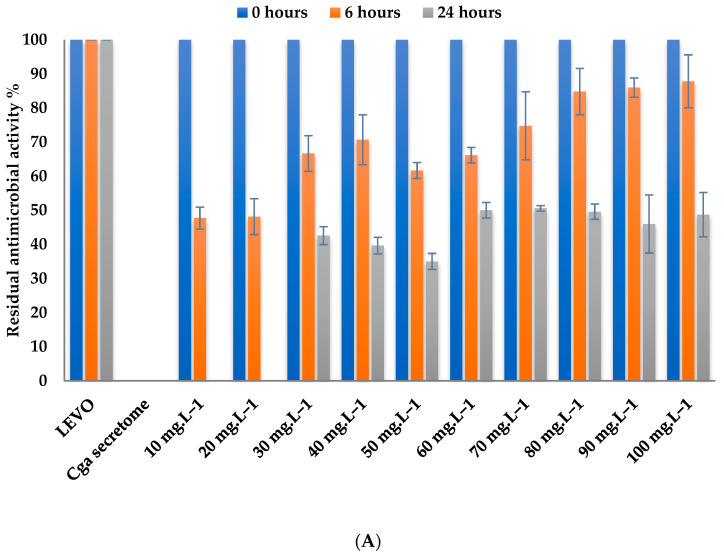

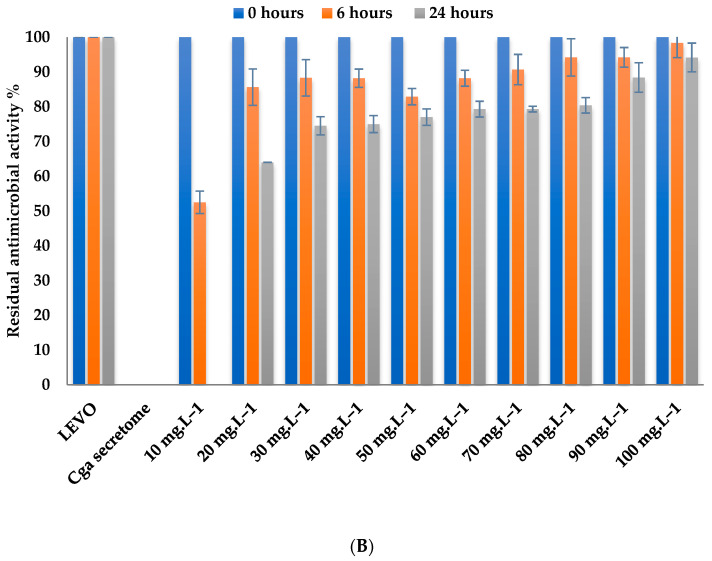
Effect of levofloxacin concentration on its biotransformation using 5 U mL^−1^ of laccase-like activity containing free (**A**) and immobilized (**B**) *Coriolopsis gallica* secretome after 6 and 24 h of incubation at 30 °C and pH 5. ■ Antimicrobial activity at T0. ■ Residual antimicrobial activity after 6 h of incubation. ■ Residual antimicrobial activity after 24 h of incubation. Chemical controls (antibiotic in the culture medium) were performed for each antibiotic concentration and were selected as the reference (100% of residual antimicrobial activity for LEVO and 0% of residual antimicrobial activity for *Cga* secretome). The error bars represent the standard deviation from the mean of the measured parameter across three experimental replicates.

The effect of levofloxacin concentration on levofloxacin biotransformation was studied by incubating the secretome with different concentrations of the antibiotic (10–100 mg L^−1^) at 30 °C and pH 5 for 24 h (Figure 2A). After 6 h, only 10 and 20 mg L^−1^ levofloxacin reached the maximal degradation yield of 50%. After 24 h, the antibiotic biotransformation reached 100% for low antibiotic concentrations (10 and 20 mg L^−1^), while the biotransformation did not exceed 50% for the higher concentrations of levofloxacin at this time. Thus, we selected a mid-way concentration of antibiotic corresponding to 50 mg L^−1^ of levofloxacin to test the possibility of improving the process to higher than 50%.

Figure 2B shows a 50% biotransformation rate and a complete biotransformation (100%) of 10 mg L^−1^ of antibiotic after 6 and 24 h of incubation, respectively. The percentage of biotransformation dropped significantly from 20 mg L^−1^ to 100 mg L^−1^. For instance, biotransformation of 100 mg L^−1^ is only 5.8% after 24 h of incubation. By comparing the results obtained with the free secretome and the immobilized one, these results could suggest that immobilization of the secretome in the alginate beads hinders their efficiency.

#### 3.4.2. Effect of Mediator Concentration

**Figure 3:**
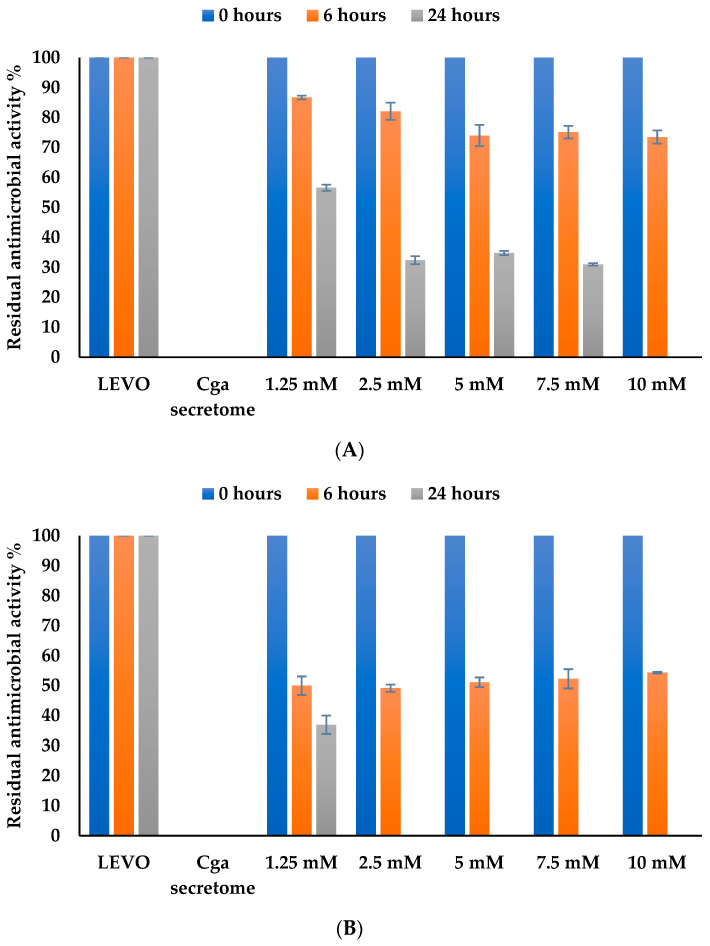
Effect of HBT concentration on the biotransformation of levofloxacin by (**A**) free and (**B**) immobilized secretome after 24 h of incubation at 30 °C and pH 5. ■ Antimicrobial activity at time = 0 h. ■ Residual antimicrobial activity after 6 h of incubation. ■ Residual antimicrobial activity after 24 h of incubation. Chemical controls (antibiotic and the culture medium) were performed for each HBT concentration and were selected as the reference LEVO and *Cga* secretome (100% of residual antimicrobial activity for LEVO and 0% of residual antimicrobial activity for *Cga* secretome). The error bars represent the standard deviation from the mean of the measured parameter across three experimental replicates.

The effect of HBT was tested over the range of 1.25 to 10 mM (Figure 3A,B). After 6 h, percentage of biotransformation was higher at higher concentrations of HBT but still did not exceed 30%. After longer incubation times, the residual antimicrobial activity was only 56% at low HBT concentrations (1.25 mM), while for mid-range HBT concentrations (2.5, 5, and 7.5 mM), the residual antimicrobial activity exceeded 30%. Levofloxacin biotransformation was 100% after 24 h of incubation in the presence of the highest concentration of HBT (10 mM), demonstrating that the presence of HBT mediator is critical and that biotransformation by the free secretome of *C. gallica* is greatly improved by using the artificial mediator. For further experiments, we used a concentration of 2.5 mM HBT to limit artificial mediator spreading.

For the immobilized secretome (Figure 3B), there was no difference in the biotransformation at 6 h of incubation (50% biotransformation). However, after 24 h of incubation, we obtained 100% biotransformation at HBT concentrations ranging from 2.5 mM to 10 mM, while residual antimicrobial activity was around 36% at a HBT concentration of 1.25 mM. For the following experiments, we selected a concentration of 2.5 mM HBT to limit the concentration of artificial mediator in the biotransformation process.

#### 3.4.3. Effect of Enzyme Concentration on Levofloxacin

**Figure 4:**
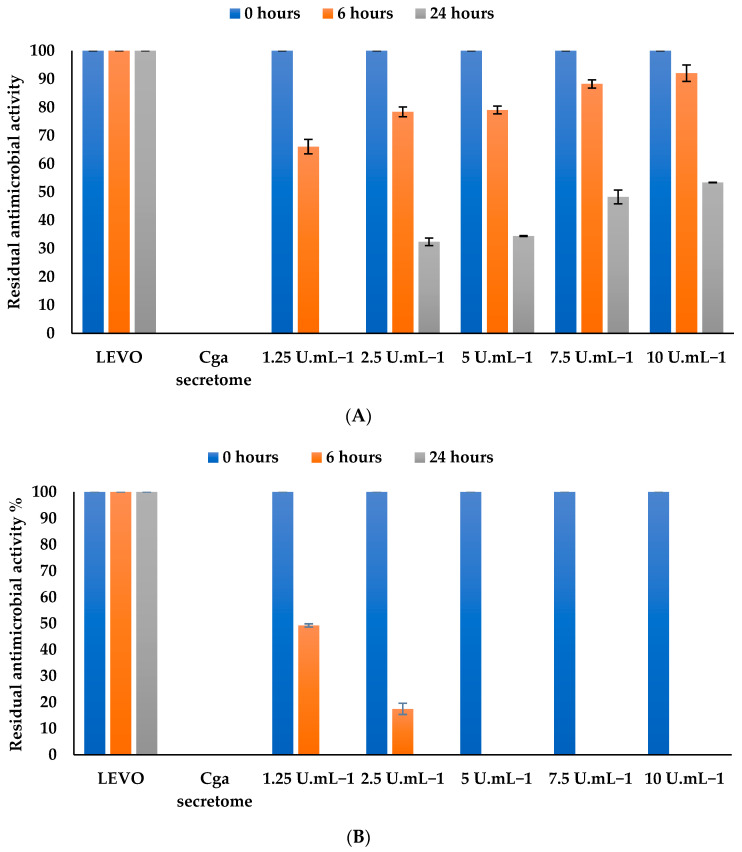
Effect of (**A**) free and (**B**) immobilized secretome concentration on levofloxacin biotransformation after 24 h of incubation at 30 °C and pH 5. ■ Antimicrobial activity at time = 0 h. ■ Residual antimicrobial activity after 6 h of incubation. ■ Residual antimicrobial activity after 24 h of incubation. Chemical controls (antibiotic and the culture medium) were performed for each secretome concentration and were selected as the reference LEVO and *Cga* secretome (100% of residual antimicrobial activity for LEVO and 0% of residual antimicrobial activity for *Cga* secretome). The error bars represent the standard deviation from the mean of the measured parameter across three experimental replicates.

The concentration of enzymes is a key parameter for reaction kinetics. Several cell-free secretome concentrations from 1.25 to 10 U mL^−1^ of laccase-like activity were tested (Figure 4A). For short or long periods of incubation (6 or 24 h), a low enzymatic concentration, i.e., 125 U mL^−1^, afforded more efficient biotransformation than the high concentration (10 U mL^−1^), which confirms that the balance between enzyme concentration and HBT concentration is critical for levofloxacin biotransformation performance. For instance, when using 1.25 U mL^−1^ of secretome, residual antimicrobial activity reached around 67% at 6 h down to 0% at 24 h of incubation.

A complete biotransformation (100%) was observed, from 1.25 U mL^−1^ to 10 U mL^−1^ of immobilized secretome after 24 h of incubation (Figure 4B). These results confirm that entrapment of the enzyme in alginate beads protects the enzyme against risks of inactivation and promotes the efficiency of biotransformation. For the following experiments, we selected a laccase-like activity of 2.5 U mL^−1^ of immobilized secretome.

#### 3.4.4. Effect of Temperature

**Figure 5:**
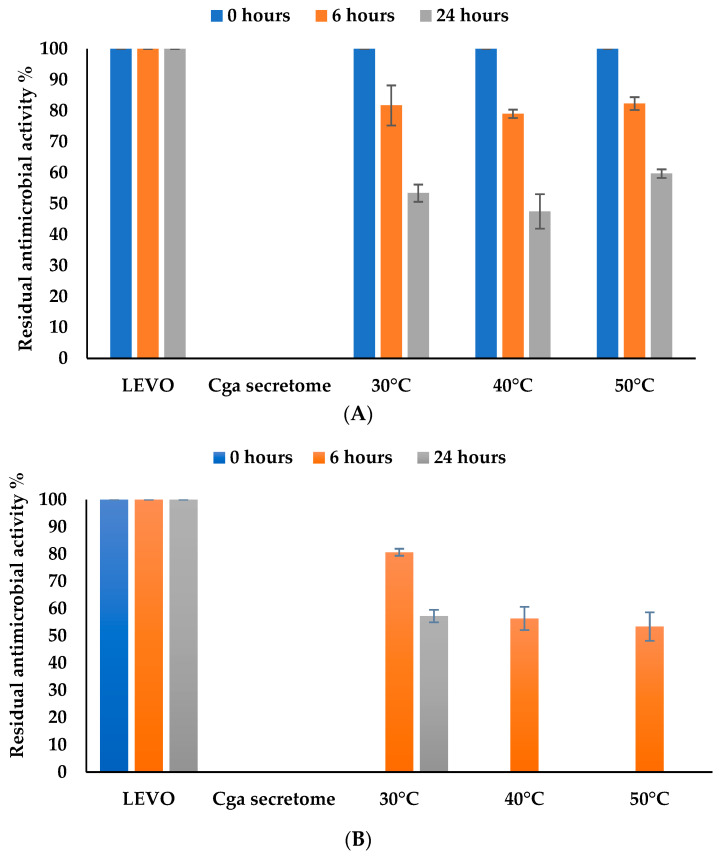
Effect of temperature on levofloxacin biotransformation by (**A**) free and (**B**) immobilized secretome after 24 h of incubation at pH = 5. ■ Antimicrobial activity at time = 0 h. ■ Residual antimicrobial activity after 6 h of incubation. ■ Residual antimicrobial activity after 24 h of incubation. Chemical controls (antibiotic and the culture medium) were performed for each temperature value and were selected as the reference LEVO and *Cga* secretome (100% of residual antimicrobial activity for LEVO and 0% of residual antimicrobial activity for *Cga* secretome). The error bars represent the standard deviation from the mean of the measured parameter across three experimental replicates.

As in most enzymatic reactions, the temperature of the LEVO biotransformation process is also crucial, as the efficiency of the enzymes is directly linked to reaction temperature. Here we tested three process temperatures from 30 °C to 50 °C. The optimal temperature for the biotransformation of levofloxacin by free secretome (Figure 5A) was 40 °C, which led to a residual antimicrobial activity of around 50% after 24 h of incubation. Then, the biotransformation decreased following the temperature activity of *C. gallica* laccases as the main enzymes described in its secretome [32,47].

Results for the immobilized secretome (Figure 5B) showed that levofloxacin biotransformation reached 100% at higher temperatures (40 °C and 50 °C), while the residual antimicrobial activity of levofloxacin was around 42.8% after 24 h of incubation at 30 °C. These results confirm that encapsulation of *C. gallica* secretome in alginate beads confers greater stability to high temperatures for the immobilized secretome. Indeed, comparison of the temperature effect on levofloxacin biotransformation by free versus immobilized secretome showed that immobilization improved the heat stability of the laccase and therefore the biotransformation percentage of levofloxacin.

#### 3.4.5. Effect of pH

**Figure 6:**
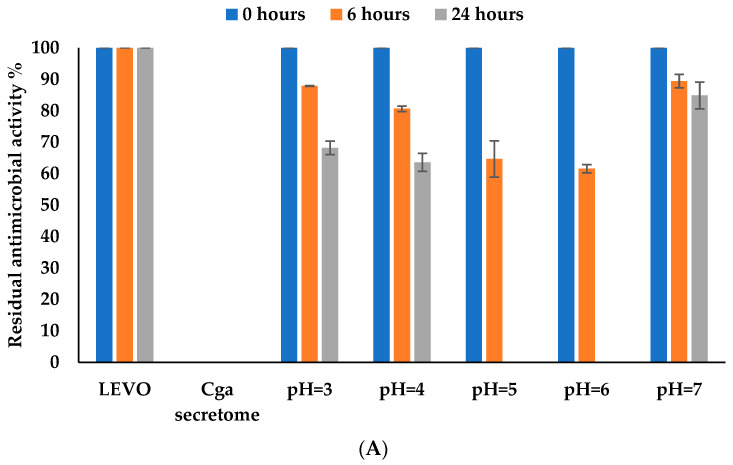

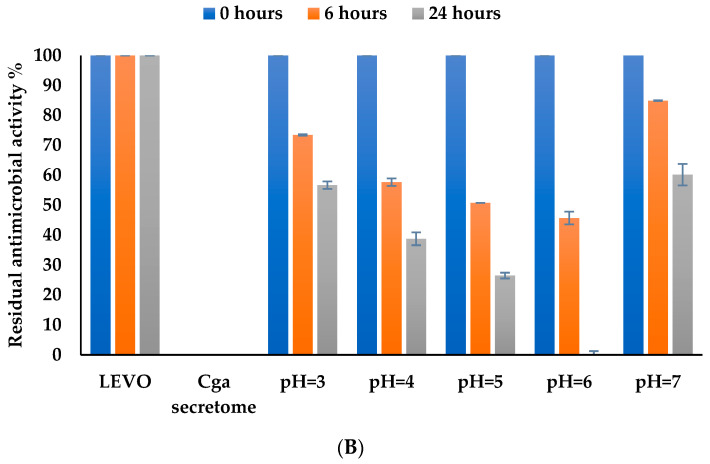
Effect of pH on levofloxacin biotransformation of by (**A**) free and (**B**) immobilized secretome after 24 h of incubation at 30 °C. ■ Antimicrobial activity at time = 0 h. ■ Residual antimicrobial activity after 6 h of incubation. ■ Residual antimicrobial activity after 24 h of incubation. Chemical controls (antibiotic and the culture medium) were performed for each pH value and were selected as the reference LEVO and *Cga* secretome (100% of residual antimicrobial activity for LEVO and 0% of residual antimicrobial activity for *Cga* secretome). The error bars represent the standard deviation from the mean of the measured parameter across three experimental replicates.

The biotransformation percentage of levofloxacin by the free *C. gallica* secretome (Figure 6A) reached a maximum of 35% and 100% at pH 5 and pH 6 after 6 and 24 h of incubation, respectively, followed immediately by a stunning decrease observed at pH 7 where only 20% of biotransformation was reached. Indeed, it has been shown that many fungal laccases require acidic pH values to achieve optimal activity [48,49]. pH variation can therefore affect the properties of the laccase activity as well as the substrates and consequently affect the efficiency of the biotransformation process.

Results for the immobilized secretome (Figure 6B) showed that levofloxacin biotransformation reached a maximum value of 100% at pH 6 and 73% at pH 5 after 24 h of incubation. At pH 7, the percentage of biotransformation of levofloxacin was 40%, i.e., about 20% higher than with the free secretome. These results confirm that the immobilized *C. gallica* secretome presents significant stability at acidic pH compared to the free secretome.

Entrapment of the secretome into Ca-alginate beads improved levofloxacin biotransformation performance across the range of pH values tested, probably because the enzyme gained stability through immobilization. Entrapment into alginate beads improves catalytic stability [50,51].

### 3.5. UHPLC–UV–MS Analyses of Coriolopsis gallica Secretome Extracts, or Laccase, in the Presence of Levofloxacin for the Dereplication of Antibiotic Degradation Products

**Figure 7:**
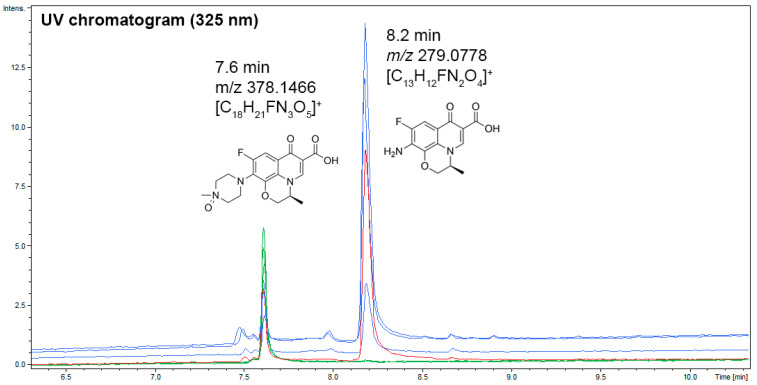
UV chromatograms (325 nm) of levofloxacin degradation products at 30 °C with tartaric acid buffer (pH 5) after 24 h of incubation [in green, levofloxacin + tartaric acid as controls (*n* = 3); in blue, Coriolopsis gallica secretome + levofloxacin + BHT 2.5 mM + tartaric acid (pH 5) (*n* = 3); in red, laccase + levofloxacin + BHT 2.5 mM + tartaric acid buffer (pH 5) (*n* = 1)].

Analysis of UV chromatograms (325 nm) of levofloxacin in the presence only of tartaric acid (pH 5) after 24 h of incubation resulted in the levofloxacin total oxidation in N-oxide levofloxacin as a unique degradation product (Figure 7). The retention time of this product (6.2 min), its *m*/*z* 378.1466 for the formula [C_18_H_21_FN_3_O_5_]^+^, and its fragmentation pattern are consistent with N-oxide levofloxacin (CAS number 117678-38-3) already detected in previous studies (Devi and Chandrasekhar, 2009; Ben Ayed et al., 2022) [32,52]. When levofloxacin is allowed to incubate with *Coriolopsis gallica* secretome in the same conditions as previously, another degradation product at 8.2 min is detected. This retention time (8.2 min), *m*/*z* 279.0778 consistent with the formula [C_13_H_12_FN_2_O_4_]^+^, and the fragmentation pattern confirmed the structure of levofloxacin with an amine in place of the piperazine cycle (CAS number 151250-74-7). Both degradation products were detected when laccase was incubated with levofloxacin in the same conditions as previously. This result suggests that the laccase contributes to levofloxacin degradation in *C. gallica* secretome.

## 4. Discussion

*C. gallica* is considered a promising candidate for bioremediation applications, as it has the ability to degrade a wide range of phenolic compounds, many of which are potential pollutants in industrial wastewaters [53,54]. *C. gallica* secretes various ligninolytic enzymes that play a key role in the biotransformation of these recalcitrant aromatic compounds, and studies have already tested its efficiency in degrading fluoroquinolones [32]. Ben Ayed et al. (2022) showed that the secretome of *C. gallica* presents a high levofloxacin removal rate of 25% after 10 days of culture. To improve the biotransformation rate, a proteomic analysis coupled with enzymatic assays of the secretome was proposed and demonstrated that the main enzyme involved in this process was a putative laccase [32]. Here, to further understand and optimize the biotransformation process, we conducted a detailed investigation into the effect of various physical–chemical parameters on the efficiency of levofloxacin biotransformation by free and immobilized secretome of this fungus. The cell-free secretome was immobilized using 2% sodium alginate followed by the addition of 2% CaCl_2,_ [41,55], yielding an immobilization efficiency of approximately 98%. In the same immobilization conditions, Daâssi et al. (2014) obtained an immobilization efficiency of around 88% [41]. Another experiment used 4% (*w*/*v*) sodium alginate in 2% (*w*/*v*) calcium chloride solution and obtained about 89% efficiency of immobilization of *T. versicolor* laccase [56].

In order to determine the effect of physical–chemical parameters on the process of levofloxacin biotransformation by free and immobilized *C. gallica* secretome, we analyzed the effect of different levofloxacin concentration, secretome activity, HBT mediator concentration, temperature, and pH of the reaction. We showed that 5 U mL^−1^ of free *C. gallica* secretome was able to biotransform 50% of a 50 mg L^−1^ concentration of levofloxacin after 24 h of incubation, while 5 U g^−1^ of immobilized secretome was able to totally biotransform 100% of a 10 mg L^−1^ concentration of the antibiotic. In general terms, the cell-free secretome demonstrated more efficient biotransformation. This difference might have been due to decreased gel porosity, high viscosity of the beads entrapping the enzyme, or substrate diffusion limitation [57,58].

Our investigation into the effect of HBT on the biotransformation of levofloxacin showed that the HBT mediator plays a critical role in enhancing the biotransformation capabilities of the cell-free secretome, whereas the immobilized secretome system exhibited different responses to HBT concentrations. At the lower 1.25 mM HBT concentration, cell-free experiments and immobilized secretome achieved only 50% biotransformation of levofloxacin, whereas HBT concentrations ranging from 2.5 mM to 10 mM resulted in full 100% biotransformation after 24 h of incubation. The immobilization process may have provided a favorable environment for laccase-like enzyme-mediated biotransformation, particularly when coupled with optimal HBT concentrations. However, even if high concentrations of the HBT mediator (e.g., 10 mM) can lead to 100% biotransformation of levofloxacin, they may also have a detrimental effect on the activity of the secretome. High concentrations of HBT can potentially cause inactivation of the laccase-like enzymes, leading to a decrease in the overall biotransformation efficiency [59]. Here we chose to focus on the moderate HBT concentrations (2.5 mM) for further experiments, as they provided a balance between enhanced biotransformation and optimal laccase-like activity. Ashrafi et al. (2020) evaluated the biotransformation of the two fluoroquinolone antibiotics enrofloxacin and flumequine in the presence of HBT mediator, and found that the best percentage of biotransformation was achieved at 2 mM HBT [60]. The literature contains ample research on the determination of optimum HBT concentrations to obtain maximum decolorization using a laccase-HBT system [60,61]. For instance, biotransformation percentage decreased at high concentrations of HBT for free and immobilized laccase, probably because the nitroxide radical resulting from laccase-driven oxidation of HBT could have a toxic effect against laccase. However, our results were consistent with those of Mechichi et al. (2006), who observed no toxic effect of 0.125 and 2.5 mM HBT on decolorization of Remazol Brilliant Blue R dye, whereas 5 mM HBT inhibited the decolorization process and significantly decreased percentage decolorization in that study [62,63].

The third parameter tested here is the effect of laccase like-activity on biotransformation assays from 1.25 to 10 U mL^−1^. Our results showed that for free-form secretome, low enzyme concentrations, i.e., 1.25 U mL^−1^ and 2.5 U mL^−1^, were more efficient than the high concentration (10 U mL^−1^), whereas for the immobilized-form secretome, results showed 100% biotransformation at 1.25 U mL^−1^ up to 10 U mL^−1^. These results confirm that the balance between enzyme concentration and HBT concentration is a critical parameter for levofloxacin biotransformation. Excessive oxidation of HBT due to a high concentration of laccase-like activity can result in the formation of side-products or byproducts that may inactivate or degrade the HBT mediator [62,63]. This inactivation can, in turn, limit the availability of mediator for the laccase-like-catalyzed biotransformation reactions, thereby reducing the overall efficiency of the process [59]. However, immobilization of the secretome may have protected the enzymes against high HBT concentrations due to the physical separation between the enzymes and the inhibitory effect of the oxidized HBT, thus protecting the overall laccase-like catalytic activity. Among the other parameters investigated, temperature also played a key role in the levofloxacin biotransformation. The optimal temperature for biotransformation of the free-form secretome was 40 °C, which led to around 50% biotransformation, whereas for the immobilized secretome, high temperatures (40–50 °C) enabled 100% biotransformation. Similar results were reported by [64], showing a maximum activity at 45 °C for free-form laccase and 55 °C for immobilized laccase. This increased efficiency at a higher temperature may be explained by the fact that, due to thermal denaturation of the tertiary structure, especially a disruption around the copper-binding sites, the laccase loses activity [65], whereas immobilized laccase is more stable against denaturing factors such as temperature [66,67]. These results indicated that immobilization of the secretome had a significant stabilizing effect against temperature, which is a clear advantage for potential bioremediation applications. The effect of pH on levofloxacin biotransformation by free and immobilized secretome was tested by carrying out the reaction at values ranging from pH 3 to pH 7. Biotransformation reached a maximum value of 100% at pH 5–6 for the immobilized secretome, whereas for the free secretome, the best percentage of biotransformation was at pH 5. Previous work has shown that fungal laccases require acidic pH values to achieve optimal activity [48,49,68]. pH variation therefore affects the properties of the laccase as well as the properties of the substrates, and consequently affects the efficiency of biotransformation. In conclusion, our best result of biotransformation was about 70%, i.e., slightly higher than that obtained with the laccase form *Pycnoporus cinnabarinus* [69,70], which reached 50% of biotransformation in the same conditions. This shows the efficiency of *Cga*Lac1 for future application in antibiotic bioremediation.

To identify the transformation products generated by the secretome and the purified laccase of *C. gallica*, as well as to confirm the role of laccase in the biotransformation of levofloxacin, we conducted a mass spectrometry analysis on samples treated with laccase or secretome, with or without the addition of levofloxacin, and incubated for 24 h. The results demonstrated that levofloxacin was completely transformed into N-oxide levofloxacin. This finding aligns with previous studies by Devi and Chandrasekhar (2009) [52], Czyrski et al. (2019) [71], and Ben Ayed et al. (2022) [32]. Moreover, when levofloxacin was treated with secretome or purified laccase, another byproduct was detected, indicating a structural modification of levofloxacin where an amine replaces the piperazine cycle. Notably, Foti et al. (2022) reported that levofloxacin was also transformed into N-oxide when exposed to sunlight-driven advanced oxidation processes (AOPs), suggesting that specific functional groups, particularly the piperazine ring, are primary sites for the degradation of quinolones [72]. This process is linked to a loss of the molecule’s antibacterial properties. However, in another study involving *Trichoderma* species (*T. asperellum* and *T. harzianum*), the major biotransformation product of ofloxacin was identified as OFL2 [C18H20FN3O5] with a *m*/*z* of 378.1465, indicative of an oxygen atom addition likely resulting from a hydroxylation process. The same study also detected three additional transformation products at *m*/*z* 348.1350 (N-desmethyl-ofloxacin), 318.1612 (decarboxylated ofloxacin), and 364.1573 (dehydrogenated ofloxacin). In a recent study investigating the biotransformation of levofloxacin by the microalga *Scenedesmus obliquus* in synthetic saline wastewater, six metabolic byproducts were identified through GC–MS analysis following the microalgal degradation process [73]. These byproducts resulted from various chemical modifications of levofloxacin, including decarboxylation, hydroxylation, dehalogenation, side chain breakdown, and ring cleavage by *S. obliquus*. A comparative study conducted by Čvančarová et al. (2015) examined the biotransformation of ciprofloxacin (CIP), norfloxacin (NOR), and ofloxacin (OFL) by five species of basidiomyces white-rot fungi: *Irpex lacteus*, *Panus tigrinus*, *Dichomitus squalens*, *Trametes versicolor,* and *Pleurotus ostreatus* [31]. The identified metabolites showed alterations exclusively in the piperazine moiety, with no modifications detected in the aromatic core of the fluoroquinolones. Changes to the piperazine ring included substitutions such as acetylation and formylation, as well as partial or complete decomposition.

## 5. Conclusions

Ben Ayed et al. (2022) previously identified *Cga*Lac1 as the main enzyme of the proteome in cultures supplemented with levofloxacin, and hypothesized that this enzyme could be potentially involved in the biotransformation of levofloxacin. Through mass spectrometry analysis, the end-product of its biotransformation was identified as the N-oxide levofloxacin, thus confirming the hypothesis of Ben Ayed et al. The generated metabolite when using the *C. gallica* secretome and the purified laccase 1 was similar to that previously shown for levofloxacin biotransformation by the fungus *C. gallica* [32]. Further studies are planned to investigate heterologously produced laccase 1 and test whether this enzyme can further improve the biotransformation of levofloxacin and other fluoroquinolones.

## Figures and Tables

**Table 1:** Summary of the purification of *Cga*Lac1.

Step	Total Activity (U)	Protein Quantity (mg)	Specific Activity (U/mg)	Purification Factor	Activity Recovery (%)
Culture medium	1471	24.9	59	1	100
Concentration with PEG	1375	13.3	103	1.74	93.4
Ammonium sulfate precipitation	403	2.2	184	3.12	27.4
Q-Sepharose	270	0.9	300	5.1	18.3

## Data Availability

The original contributions presented in this study are included in the article. Further inquiries can be directed to the corresponding authors.
